# Programmed Cell Death in Health and Disease

**DOI:** 10.3390/cells10071765

**Published:** 2021-07-13

**Authors:** Lara Gibellini, Loredana Moro

**Affiliations:** 1 Department of Medical and Surgical Sciences for Children and Adults, University of Modena and Reggio Emilia, 41121 Modena, Italy; 2 National Research Council, Institute of Biomembranes, Bioenergetics and Molecular Biotechnologies, 70126 Bari, Italy

Programmed cell death is a conserved evolutionary process of cell suicide that is central to the development and integrity of eukaryotic organisms. Dysregulation of this program is associated with a wide range of diseases, including developmental disorders, immunodeficiency, autoimmune diseases, neurodegeneration, and cancer [1]. The term “apoptosis”, the first type of programmed cell death to be described [2], was introduced in 1972 by Professor John Kerr et al. as a complementary but opposite mechanism to mitosis in the regulation of an animal cell population: “Because of its important kinetic significance, we suggest that it be called ‘apoptosis’, a word used in Greek to describe the ‘falling off of petals from flowers or leaves from trees.’” [2].

The first evidence of programmed cell death came from studies performed on the nematode *C. elegans*: adult nematodes have an invariant number of cells: 959 somatic cells for a “female” (in fact, a hermaphrodite able to produce eggs and sperm) and 1031 for an adult male [3]. Forty years ago, Sulston, Horvith and Thomson described the fate of every cell in *C. elegans* from embryo to adult and discovered that the death of 131 cells followed by their engulfment by the neighboring cells led to the generation of the hermaphrodite with 959 cells. This discovery proved that apoptosis was genetically programmed, not fortuitous. Mutations affecting apoptosis in nematodes were also described [4]. Since this discovery, the molecular mechanisms of apoptosis have been characterized in detail in human cells, but a new level of complexity has begun to emerge indicating that, besides apoptosis, different programmed cell death programs may co-exist in the same cell and that molecular effectors of self-destruction programs may also perform additional functions essential for cell survival [1]. 

In “Programmed Cell Death in Health and Disease” (a Special Issue of *Cells*), a panel of leading scientists provides an exhaustive overview of different facets of programmed cell death programs in a variety of cell types and pathophysiological conditions. Collectively, the 12 contributions in “Programmed Cell Death in Health and Disease” (two original articles and 10 reviews) highlight the main molecular and therapeutic aspects of cell suicide programs in neurodegenerative diseases, inflammation, infection, ectopic calcification, tumor chemoresistance and anti-cancer therapy. 

Programmed cell death pathways are hyperactive in various neurodegenerative disorders including Alzheimer’s, Parkinson’s and Huntington’s diseases [5]. In these disorders, cell suicide programs are pathogenic, which means that targeting them might slow neurodegeneration or mitigate symptoms. In their research paper, Gil et al. [6] assessed the effect of fingolimod phosphate in a model of oxidative damage induced in neuronal cell cultures by menadione (vitamin K3). Fingolimod phosphate, a drug with antioxidant properties used to treat multiple sclerosis, promoted glycolysis and the pentose phosphate cycle, and it exerted a beneficial effect on the menadione-induced oxidative damage by improving mitochondrial activity and restoring oxidative balance in the neuronal cells. This suggests that this drug may represent a therapeutic tool to slow the progression of neurodegeneration. 

In their review article, Atlante and colleagues [7] discuss the beneficial action of some natural products in providing protection against mitochondrial dysfunction, oxidative damage, toxicity of β-amyloid and Tau, and apoptotic cell death in Alzheimer’s disease. Indeed, as discussed by the authors, nutraceuticals can induce epigenetic changes of gut microbiota, which, in turn, may prevent or slowdown the formation and aggregation of toxic proteins in the brain. 

Besides neurodegeneration, the dysregulation of apoptotic signaling pathways and excessive apoptosis are implicated in the development and progression of other pathological conditions such as parasitic and viral infection, and ectopic calcification, a pathological state that worsens the quality of life of aged patients by affecting the cardiovascular system (vessels and valves) and joints [8]. In their review, Boraldi et al. [9] present an overview of ectopic calcification mechanisms in cartilage and vascular tissue, focusing on the interplay between apoptosis and mineralization. Notably, during extraosseus calcification, apoptotic bodies may function as nucleation sites for crystal deposition. The review also discusses the changes in several molecular effectors in cartilage and vascular apoptosis, including death receptors, cytokines and growth factors, reactive oxygen and nitrogen species, microribonucleic acids and long non-coding RNAs, which may be involved in the pathogenic mechanisms of ectopic calcification. 

Sena-dos-Santos and colleagues [10] review the role of programmed cell death in malaria, a parasitic disease caused by the protozoa of the *Plasmodium* genus that still affects millions of people worldwide (https://www.who.int/health-topics/malaria; accessed on 7 July 2021). Specifically, they discuss several cell death programs triggered by *Plasmodium* infection: apoptosis, necrosis, autophagy, pyroptosis, ferroptosis and NETosis, a form of regulated necrosis that causes intravascular inflammation. The authors also highlight the dual role of cell death in the host-protective immune response and pathogenesis of severe malaria by focusing on pathways and factors involved in malaria-triggered cell death as therapeutic targets. 

In their review, Gruevska et al. [11] summarize the current knowledge on apoptosis activation in hepatic cells induced by human immunodeficiency virus (HIV) infection, HIV-encoded viral proteins or anti-HIV/antiretroviral drugs. Their focus is on the mechanisms that lead to HIV-related caspase activation and cell death, including induction of mitochondrial dysfunction, oxidative stress, endoplasmic reticulum (ER) stress and unfolded protein response (UPR). As the authors point out, a comprehensive knowledge of the mechanisms that drive development of hepatic disease in HIV-infected people may improve their existing antiretroviral therapies and overall clinical management. 

Paolini et al. review the role of apoptosis and other forms of cell death, including necroptosis and pyroptosis, in coronavirus infections, with a special focus on cell death programs caused by the severe acute respiratory syndrome coronavirus 2 (SARS-CoV-2), which plays a dual role in mediating cell death [12]. On the one hand, SARS-CoV-2 exerts a direct cytopathic effect on infected cells; on the other, it acts indirectly by causing the hyperactivation of the immune system, which in turn culminates in the massive release of cytokines and chemokines by various cell types in a process known as “cytokine storm”. Specifically, the synergy between tumor necrosis factor (TNF) and interferon-γ (IFN-γ) triggers apoptosis, necroptosis and pyroptosis in immune cells leading to lymphopenia and monocytopenia, and therefore contributing to the immunopathogenesis of Coronavirus Disease 2019 (COVID-19). PANoptosis is the phenomenon of crosstalk among the three main cell death pathways that lead to a unique inflammatory programmed form of cell death. The authors emphasize that studying cell death during COVID-19 could improve understanding of the pathogenesis of this disease, and identify promising therapeutic options to mitigate the damage caused by a pathogenic immune response.

The role of mitochondria in cell death during inflammation was reviewed by Picca et al. [13]. The authors summarize current knowledge about the displacement of mitochondrial DNA (mtDNA) into extracellular compartments and its involvement in modulating the innate immune response. This review spans mechanisms leading to mtDNA displacement, including alterations in mitochondrial dynamics and mitophagy, to the emerging role of mtDNA unloading in priming anti-tumor immunity via immunological cell death. An interesting conclusion is that crosstalk between autophagy and apoptosis is emerging as a means to regulate cell death and survival.

Caspase-2 can have both pro-apoptotic and pro-survival regulatory functions and its role in regulating cell fate is discussed in the contribution by Vigneswara and Ahmed [14]. Caspase-2 is the most evolutionarily conserved member of the mammalian caspase family and exists in two functionally different splice variants: the pro-apoptotic caspase-2L and the short-lived anti-apoptotic, caspase-2S. The authors cover aspects of caspase-2 structure, subcellular localization, activation pathways in physiological and pathological settings, and interaction with adaptor molecules. DNA damage and other stressors, including cytotoxic drugs and reactive oxygen species, emerged as important activators of pro-apoptotic caspase-2. An additional pathway driving caspase-2 activation is represented by the multiprotein complex known as “PIDDosome”, which acts as a critical regulatory component controlling cell cycle arrest and DNA repair. However, despite the vast progress over the past years in understanding the regulation of caspase-2, full comprehension of the signaling pathways it mediates is still missing and requires additional work. 

“Evading apoptosis” is the third hallmark of malignant tumor cells [15]: unlike neurodegeneration and infection, malignant transformation is characterized by the acquisition of an intrinsic resistance to programmed cell death that is also responsible for resistance to therapeutic treatment. In their research paper, Caillot et al. [16] tested whether the generation of ROS imbalance would sensitize/resensitize multiple myeloma cells to bortezomib, a proteasome inhibitor. The authors used a NADPH oxidase or a thioredoxin reductase inhibitor and found that the combination of the inhibitor with bortezomib synergistically induced apoptosis and autophagy in multiple myeloma cell lines and primary cells. Taken together, the results suggest that modulating the ROS balance of multiple myeloma cells may prove effective for refractory post-bortezomib patients. 

In their review, Fiorcari et al. [17] discuss the prospects of specifically targeting and “re-educating” nurse-like cells to promote drug-induced apoptosis in chronic lymphocytic leukemia. Indeed, nurse-like cells are a specific population of leukemia-associated macrophages inside tissue niches that feed and protect leukemia cells from drugs and, in continuous crosstalk with leukemic cells, are reprogrammed to generate an immunosuppressive microenvironment to allow immune evasion. Thus, translational approaches designed to target nurse-like cells with drugs that deplete tumor-associated macrophages or re-educate them against leukemia may be instrumental for the next generation of immune-oncology agents. In their review, Menichini and colleagues [18] highlight the activities of the small molecules PRIMA-1 and its methylated form PRIMA-1^Met^ (APR246) in restoring wild-type P53, a tumor suppressor frequently inactivated in tumor cells. These molecules upregulate genes involved in cell-cycle control and apoptosis in p53-mutated tumor cells, resulting in apoptotic cell death. Clinical trials suggest that APR246 may have therapeutic potential in hematological malignancies. Wu and co-authors [19] provide a comprehensive overview of natural products of plant origin with therapeutic potential for cancer treatment. In general, many plant-derived anticancer drugs such as paclitaxel and vincristine are currently in use. This review summarizes current knowledge on promising phytochemicals found in plants with potential for bladder cancer treatment. 

In summary, this Special Issue is fully dedicated to addressing several aspects of programmed cell death, from tissue homeostasis to inflammation, immunity, and several pathophysiological conditions. Overall, these contributions further strengthen the essential function of programmed and other forms of cell death, in health and diseases and reinforce the concept that targeting these pathways could hold great promise for the treatment of multiple human disorders.

## Data Availability

Not applicable.
